# Food Hypersensitivity in Children Aged 0–3 Years of the Lviv Region in Ukraine: A Cross-Sectional Study

**DOI:** 10.3389/fped.2021.800331

**Published:** 2022-01-10

**Authors:** Oksana Matsyura, Lesya Besh, Olena Borysiuk, Taras Gutor, Andriana Malska, Oksana Kovalska, Olesia Besh, Olena Sorokopud, Sandor G. Vari

**Affiliations:** ^1^Department of Pediatrics, Danylo Halytsky Lviv National Medical University, Lviv, Ukraine; ^2^Allergy Department, Communal Nonprofit Enterprise “City Children's Clinical Hospital of Lviv,” Lviv, Ukraine; ^3^Department of Public Health Management, Danylo Halytsky Lviv National Medical University, Lviv, Ukraine; ^4^Department of Propaedeutic Pediatrics and Medical Genetics, Danylo Halytsky Lviv National Medical University, Lviv, Ukraine; ^5^Department of Therapy and Medical Diagnostics, Danylo Halytsky Lviv National Medical University, Lviv, Ukraine; ^6^International Research and Innovation in Medicine Program, Cedars-Sinai Medical Center, Los Angeles, CA, United States

**Keywords:** food hypersensitivity, hypersensitivity to milk, factors, questionnaire, young children

## Abstract

**Aim:** To determine the prevalence and to estimate factors associated with food hypersensitivity in young children of the Lviv region in Ukraine.

**Methods:** A prospective cross-sectional survey study was conducted between 2016 and 2017 in the Lviv region of Ukraine. A specially designed questionnaire about food hypersensitivity of young children developed and validated by M. J. Flokstra-de Blok was used after translation into the Ukrainian language. The questionnaire included 34 questions, grouped into general and detailed information. Parents of children aged 0–3 years were asked to complete the questionnaire at pre-schools and medical institutions.

**Results:** Among 4,500 distributed questionnaires, 3,214 (71%) were completed and processed. Parents reported that 25% of their young children had food hypersensitivity. According to the survey the most common agents involved in food hypersensitivity in young children were cow's milk (34%), egg (28%), and wheat (24%). Hypersensitivity to milk occurred in 50% of children in the age group of 1–2 years. Regional differences associated with food hypersensitivity were also found. Namely, in the Carpathians, there was more hypersensitivity to fish (27%) and honey (22%) than in other regions, while hypersensitivity to soy was detected mostly in Lviv City residents (8.5%). Unknown causes of food hypersensitivity were highly reported (34%) in the Carpathians.

**Conclusion:** Prevalence and some distinctiveness of food hypersensitivity revealed in four geographic and climate zones as well as in Lviv City have a considerable practical use for formulation of recommendations for children with food hypersensitivity.

## Introduction

Food hypersensitivity is the most common explanation for various adverse reactions to food products, including a large number of diseases which have similar clinical presentations but different pathogenic mechanisms (1). Prevalence of this nosology ranges from 3 to 30% (2, 3). The investigation of the real incidence of this pathology is rather challenging due to the absence of unified diagnostic schemes and the wide spectrum of similar clinical symptoms. Thus, the similarity of the clinical picture in different types of food hypersensitivity leads to many questions and discussions, although completely different pathogenic mechanisms are involved (4–6). The feature of an adverse reaction cannot always be distinguished; thus, there is often dissonance between the number of indicated cases of food hypersensitivity and identification of a certain type during epidemiological studies. This issue is especially urgent in the context of the diagnosis of food allergy, which is always characterized by a higher incidence from responses to a questionnaire than in the actual cases when objective methods of examination are applied (7). From a practical point of view, it is important to determine food hypersensitivity early and correctly, which then enables appropriate recommendations to be given and, in many cases, may prevent the development or progression of the disease (8).

In Ukraine, only a few investigations on food hypersensitivity and food allergy have been conducted. Due to small size as well as lack of uniform diagnostic and statistical methods, the general population representation is impossible. Incidence data for this group of diseases, which are detected during target investigation, are 7–10 times higher than the officially recorded numbers (9). A progressive increase in different forms of food hypersensitivity is associated with many factors, particularly malnutrition and harmful ecological conditions. Epigenetic and genetic factors, which potentially can be related to food hypersensitivity and allergy, are being thoroughly studied (1, 3). In this complex situation, the detection of risk factors for disease development is important for offering patients proper recommendations on diet, living conditions and lifestyle.

The aim of this study was to conduct an epidemiological investigation of food hypersensitivity in young children (ages: 0–3 years) of the Lviv region in Ukraine *via* a cross-sectional survey of their parents.

## Materials and Methods

### Study Design

This was a cross-sectional study.

### Ethics

Before the study, the protocol and research materials had been reviewed and approved by the Local Ethics Committee at Lviv City Children's Clinical Hospital in Ukraine (hospital was renamed in July, 2018 as Nonprofit Communal Enterprise “City Children's Clinical Hospital of Lviv”); Sep 16, 2014 No 7. The survey being voluntary, written parental consent for processing and dissemination of information was obtained.

### Participants

The study included parents of young children aged 0–3 years in the Lviv region of Ukraine. In cases when parents had more than one child of this age, separate questionnaires were filled in for each child.

### Study Questionnaire

**Figure 1:**
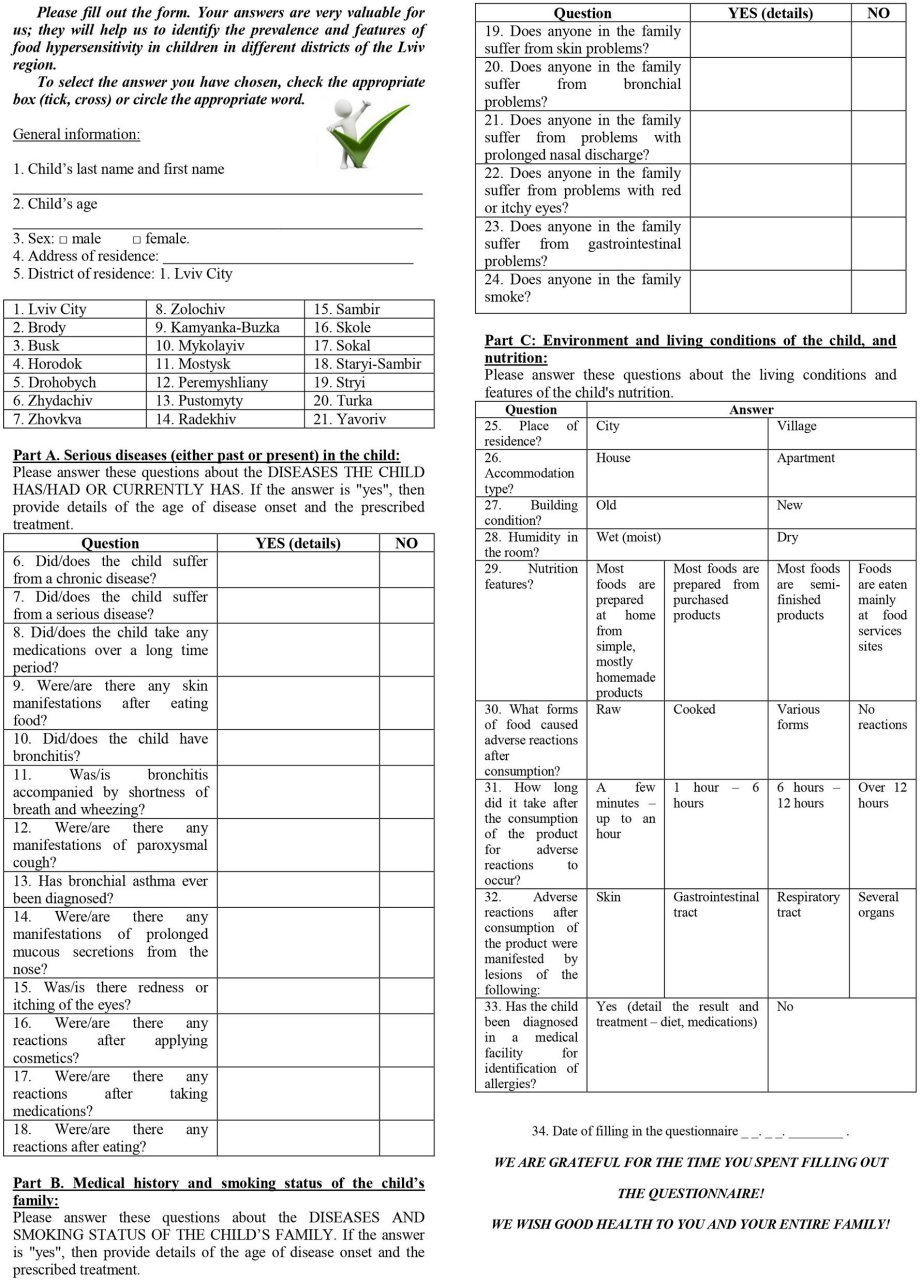
Questionnaire used to identify children with food hypersensitivity in the Lviv region.

The study was conducted using a specially designed questionnaire developed and validated by Flokstra-de Blok (10) (following translation into the Ukrainian language) that was administered to parents (Figure 1). The adaptation took place from 2016 to 2018 and after that the questioners were validated in Ukraine. The copyright was registered, received certificates from the Ukrainian Health Care Authorities, and now everyone can use the questionnaires in Ukraine (11).

The questionnaire was modified and adopted for food allergy and quality of life and had 34 questions that were grouped into four parts: *general* information and *detailed* information in parts A, B, and C (12).

The general information part of the questionnaire included questions about demographic data (age, sex) and the region where the child lived. Information about the regions where the children lived was used to categorize them into four geographic and climate zones in the Lviv region. These zones are: Forest-Grassland (Steppe), Lowland (Polesia), Carpathian foothills (Prykarpattia), and Carpathians. In general, in the Lviv region, there were a total of 20 districts and Lviv City.

The Forest-Grassland zone includes seven districts: Sokalskyi, Radekhivskyi, Zhovkivskyi, Kamyanka-Buzkyi, Buskyi, Brodivskyi, and Yavorivskyi.

The Lowland zone includes Lviv City and eight districts: Mostyskyi, Horodotskyi, Pustomytivskyi, Zolochivskyi, Peremyshlyanskyi, Sambirskyi, Mykolaivskyi, and Zhydachivskyi.

The Carpathian foothills zone includes three districts: Starosambirskyi, Drohobytskyi, and Stryiskyi.

The Carpathians zone includes two districts: Turkivskyi and Skolivskyi.

The detailed information section of the questionnaire had three parts. Part A had questions about serious diseases (either past or present) that a child had suffered. The presence of serious disease was an exclusion for participation in this prospective study. Part B asked about the medical history and smoking status of the child's family. Part C had questions about the environment and living conditions of the child, as well as nutrition and its relation to the development of the clinical symptoms of disease.

We analyzed the symptoms, complications, family history, living conditions, and nutritional habits of the children with food hypersensitivity, and compare the children with and without food hypersensitivity and added this information in **Tables 5**–**8**.

### Study Population

The study was conducted in 2016–2017 in the Lviv region in Ukraine. The questionnaire was distributed in pre-schools and medical institutions on Child Health Day. Parents with children younger than 3 years old who live in the Lviv region in Ukraine were asked by personnel of pre-schools and medical institutions to complete the questionnaire. A child could not be enrolled in the study without voluntary parental consent. All completed questionnaires were submitted to the study researchers.

### Statistical Analysis

For categorical and qualitative variables, the indices of descriptive statistics (frequency or fraction) were calculated. Frequency tables (table 2 × 2 or *n* × 2) were used to verify the significance of differences between the groups of categorical and qualitative features, and comparisons were conducted using Pearson's χ^2^ test. A *p* < 0.05 was considered to be significant. Statistical calculations were performed using the software packages RStudio v. 1.1.442 and R Commander v.2.4-4 (R Foundation for Statistical Computing, Vienna, Austria, 2020).

## Results

A total of 4,500 questionnaires were distributed to parents of young children at pre-schools and medical institutions. Responses were received for 3,267 (73%) children. After 53 (1.6%) questionnaires were excluded due to incomplete information, the remaining 3,214 questionnaires from respondents (71% of the original 4,500) were analyzed. Among these 3,214 respondents, there were 1,010 who were 0–1 years in age, 1,735 aged 1–2 years, and 469 aged 2–3 years.

There were no significant differences between the number of children for whom parents reported data in each zone of the Lviv region (*p* > 0.05), and thus, these data were comparable. The survey revealed similar numbers of boys and girls in each zone (Table 1).

**Table 1:** Sex distribution and presence of serious disease in children among five geographic and climate zones in the Lviv region.

**Zone**	**Children in zone**	**Sex**		**Serious disease in children^†^**
		**Boys**	**Girls**	
Forest-Grassland	649 (20) ^*^	330 (51)	319 (49)	26 (4.0)
Lowland	670 (21)	319 (48)	351 (52)	32 (4.8)
Carpathian foothills	673 (21)	336 (50)	337 (50)	17 (2.5)
Carpathians	607 (19)	314 (52)	293 (48)	24 (4.0)
Lviv City	615 (19)	295 (48)	320 (52)	15 (2.4)
Total number of children	3,214 (100)	1,594 (50)	1,620 (50)	114 (3.5)

* *Number (%)*.

† *Past or present diseases*.

### Serious Disease

Part A of the detailed information section of the questionnaire included questions about diseases that a child had experienced. Past or present serious disease was reported in 114 (3.5%; min 2.4%; max 4.8%; *p* = 0.09) of the 3,214 total children (Table 1).

**Figure 2:**
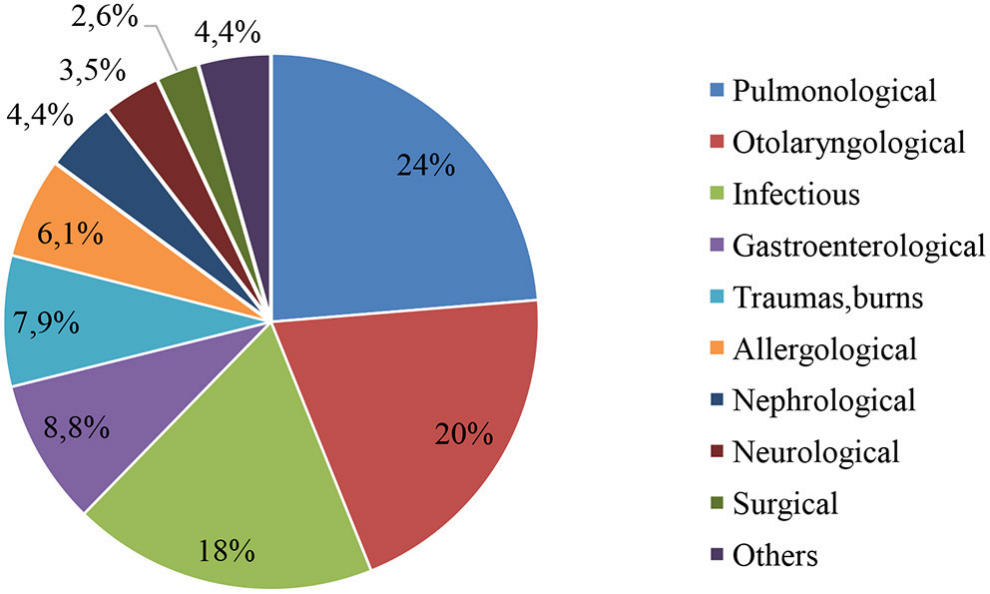
Types of previous or existing serious diseases in the respondents.

The data on the types of past or present serious diseases in children are shown in Figure 2.

Pulmonological diseases (24%, *n* = 27) were the most common type, including pneumonia (*n* = 11), laryngitis (*n* = 9), obstructive bronchitis (*n* = 5), and bronchial asthma (*n* = 2). Otolaryngological diseases were the second most common type, representing 20% (*n* = 23) of serious diseases, which included otitis (*n* = 15), tonsillitis (5), or other (*n* = 3).

Infectious diseases represented 18% (*n* = 21) of all serious diseases.

Allergological diseases were referred to as “serious diseases” in 6.1% (*n* = 7) of the respondents, and included atopic dermatitis (*n* = 4), anaphylaxis (*n* = 2), and Stevens-Johnson syndrome (*n* = 1). The course of most diseases in this group was moderate, and the children were treated in the day inpatient department or on an outpatient basis.

### Food Hypersensitivity

**Figure 3:**
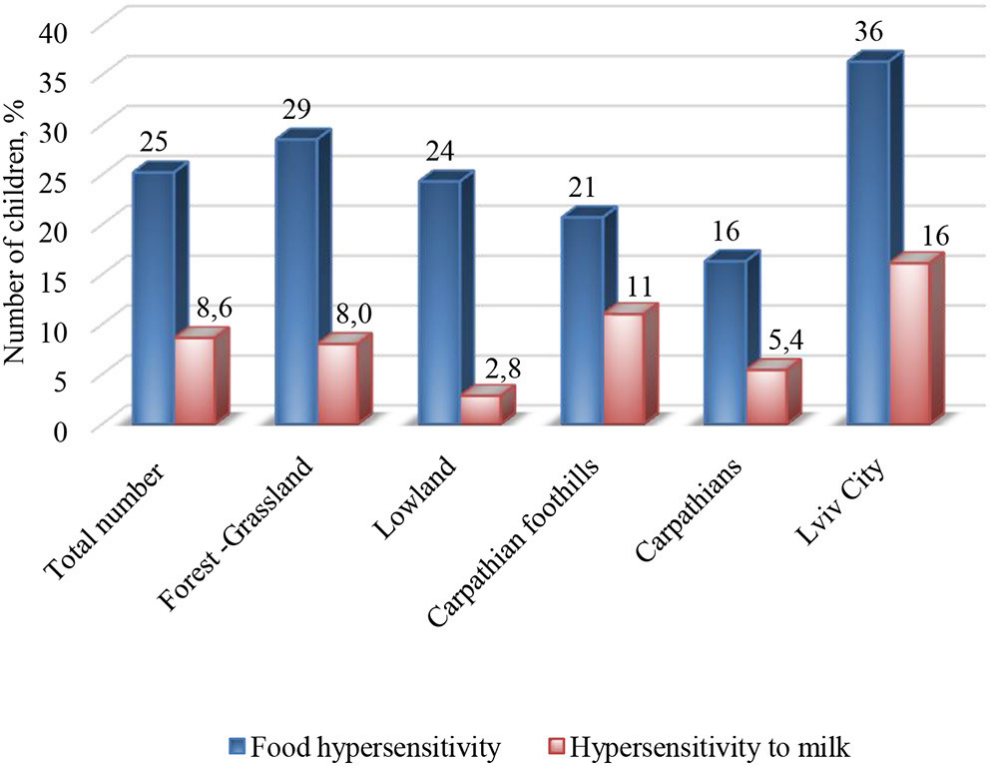
Distribution of food and milk hypersensitivity in different geographic and climate zones of the Lviv region.

Food hypersensitivity was reported in 809 (25%) children of the Lviv region, out of whom 8.6% were sensitive to milk. Distributions of food and milk hypersensitivities in different geographic and climate zones of the Lviv region are presented in Figure 3. The highest reported rate (36%) of food hypersensitivity was found in Lviv City residents, the lowest (16%) in the Carpathians. The three most common types of food hypersensitivity in these young children were milk (34%), egg (28%), and wheat (24%). Milk hypersensitivity among those with food hypersensitivity in the different geographic and climate zones of the Lviv region was as follows: Lviv City (44%), Forest–Grassland (28%), Lowland (12%), Carpathian foothills (53%), and the Carpathians (33%).

In the different geographic and climate zones of the Lviv region, food sensitivity to other products was also noted (Table 2). Namely, in the Carpathians, greater hypersensitivities to fish (27%) and honey (22%) were observed than in other regions.

**Table 2:** Food hypersensitivity (FH) of the children varies with the zone where they live.

	**Number (%) of children with food hypersensitivity in each zone**					**Total number (%) of children with FH**
	**Forest-Grassland**	**Lowland**	**Carpathian foothills**	**Carpathians**	**Lviv City**	
	185 (23)	163 (20)	139 (17)	99 (12)	223 (28)	809 (100)
**Food type**						
Milk	52 (28)	19 (12)	74 (53)	33 (33)	99 (44)	277 (34)
Egg	37 (20)	38 (23)	52 (37)	30 (30)	72 (32)	229 (28)
Wheat	33 (18)	36 (22)	38 (27)	29 (29)	54 (24)	190 (24)
Citrus	31 (17)	30 (18)	29 (21)	17 (17)	22 (9.9)	129 (16)
Cocoa	21 (11)	33 (20)	24 (17)	22 (22)	28 (13)	128 (16)
Fish	13 (7.0)	22 (14)	26 (19)	27 (27)	29 (13)	117 (15)
Nuts	8 (4.3)	26 (16)	34 (25)	20 (20)	24 (11)	112 (14)
Strawberry	11 (6.0)	25 (15)	18 (13)	15 (15)	34 (15)	103 (13)
Honey	4 (2.2)	7 (4.3)	12 (8.6)	22 (22)	11 (4.9)	56 (6.7)
Soy	2 (1.1)	4 (2.5)	1 (0.7)	0 (0)	19 (8.5)	26 (3.2)
Other	12 (6.5)	18 (11)	14 (10)	6 (6.1)	14 (6.3)	64 (7.9)
Unknown	5 (2.7)	9 (5.5)	20 (14)	34 (34)	3 (1.4)	71 (8.8)

Hypersensitivity to soy was noteworthy only in children living in Lviv City (8.5%). Unknown causes of food hypersensitivity were reported by parents for 8.8% of all children. However, this overall percentage was greatly influenced by the highest percentage of 34% that was found in the Carpathians.

The total number of assumed causative components of food hypersensitivity among these children was 1,502.

There were significant differences in hypersensitivity to food components between different geographic and climate zones (Table 3). The highest incidence of hypersensitivity to one food component was recorded in respondents of Forest-Grassland (79%). Indices were average in Lviv City and Lowland (47 and 42%, respectively), and lower in the Carpathian foothills and Carpathians (7.2 and 4%, respectively). Hypersensitivity to three or four food components was the highest in the Carpathian foothills and Carpathians (50 and 58%, respectively); however, there was also a high percentage of unidentified component in these regions (14 and 34%, respectively). This distribution may indicate that the causative component is not distinctly specified, but it is rather the parents' assumption, and thus, several most likely products are mentioned.

**Table 3:** Distribution of children among geographic and climate zones according to hypersensitivity to food components.

**Group**	**Number (%) of children with hypersensitivity to food components**					**Total number (%) of children in a group**	**Total number of food components**
	**One**	**Two**	**Three**	**Four**	**Unidentified**		
Forest-Grassland	146 (79)^*^	24 (13)	10 (5.4)	0 (0)	5 (2.7)	185 (100)	229
Lowland	69 (42)	66 (41)	19 (12)	0 (0)	9 (5.5)	163 (100)	267
Carpathian foothills	10 (7.2)	40 (29)	44 (32)	25 (18)	20 (14)	139 (100)	342
Carpathians	4 (4.0)	3 (3.0)	21 (21)	37 (37)	34 (34)	99 (100)	255
Lviv City	105 (47)	58 (26)	43 (19)	14 (6.3)	3 (1.4)	223 (100)	409
Total	334 (41)	191 (24)	137 (17)	76 (9.4)	71 (8.8)	809 (100)	1,502

* *Percentage of children with hypersensitivity to food components was calculated according to the equation below: Forest-Grassland group has 185 respondents (100%). Hypersensitivity to one food component is present in 146 responsents. Thus, 146/185 × 100% = 79%*.

There were no reliable differences between groups in terms of age and its possible relation to the development of food hypersensitivity to milk (Table 4).

**Table 4:** Distribution of children by age and food hypersensitivity to milk.

**Group**	**Number (%) of children with this age at the time of the questionnaire, years**			**Total number (%) of children**
	**0–1**	**1–2**	**2–3**	
**Without signs of milk hypersensitivity**	921 (29)	1,592 (50)	424 (13)	2,937 (91)
Boys	450 (14)	807 (25)	195 (6.1)	1,452 (45)
Girls	471 (15)	785 (24)	229 (7.1)	1,485 (46)
**With signs of milk hypersensitivity**	89 (2.8)	143 (4.4)	45 (1.4)	277 (8.6)
Boys	48 (1.5)	71 (2.2)	23 (0.7)	142 (4.4)
Girls	41 (1.3)	72 (2.2)	22 (0.7)	135 (4.2)
Total	1,010 (32)	1,735 (54)	469 (15)	3,214 (100)

The age distribution of milk hypersensitivity shows that signs of the disease had already developed in the first year of life in one-third of young participants (32%), but that the highest percentage (54%) was observed in the age group of 1–2 years. However, after 2 years of age milk hypersensitivity was found in only 15%. Thus, by the age of 2 years signs of food hypersensitivity to milk were detected in 86% of children.

### Physical Symptoms and Complications

The responses to part A of the detailed information section of the questionnaire provided information about respiratory symptoms and diseases, as well as cutaneous and mucosal problems (Table 5).

**Table 5:** Distribution of children among geographic and climate zones and presence of physical symptoms and complications^*^.

**Symptoms and problems**	**Number (%) of respondents in each zone**					**Total number (%) of participating children** ***p*** **value**		**Number (%) of respondents with FH**		
	**Forest-Grassland**	**Lowland**	**Carpathian foothills**	**Carpathians**	**Lviv City**			**Indicated symptom or problem is present**	**Indicated symptom or problem is absent**	***p* value**
Bronchitis	262 (40)	171 (26)	313 (47)	186 (31)	246 (40)	1,178 (37)	<0.001	325 (28)	484 (24)	0.018
Breathlessness and wheezing	109 (17)	49 (7.3)	111 (17)	91 (15)	74 (12)	434 (14)	<0.001	113 (26)	696 (25)	0.698
Paroxysmal cough	120 (19)	111 (17)	107 (16)	106 (18)	45 (7.3)	489 (15)	<0.001	143 (29)	666 (24)	0.028
Bronchial asthma	4 (0.6)	12 (1.8)	22 (3.3)	10 (1.6)	27 (4.4)	75 (2.3)	<0.001	24 (32)	785 (25)	0.213
Pollinosis	44 (6.8)	40 (6.0)	50 (7.4)	67 (11)	24 (3.9)	225 (7.0)	<0.001	80 (36)	729 (24)	<0.001
Reddening and itching of eyes	20 (3.1)	13 (1.9)	27 (4.0)	36 (5.9)	13 (2.1)	109 (3.4)	<0.001	40 (37)	769 (25)	0.007
Urticaria	118 (18)	133 (20)	122 (18)	100 (17)	113 (18)	586 (18)	0.650	217 (37)	592 (23)	<0.001
Reactions to cosmetics	36 (5.5)	44 (6.6)	44 (6.5)	41 (6.8)	60 (9.8)	225 (7.0)	0.045	64 (28)	745 (35)	0.274
Contact dermatitis	62 (9.6)	15 (2.2)	133 (20)	71 (12)	2 (0.3)	283 (8.8)	<0.001	104 (37)	705 (24)	<0.001
Reactions after using medications	68 (11)	64 (9.6)	157 (23)	73 (12)	56 (9.1)	418 (13)	<0.001	112 (27)	697 (25)	0.448

* *Based on analysis of detailed information from the questionnaire (part A)*.

In the children, bronchitis was diagnosed in 37% (*n* = 1,178; min 26; max 47; *p* < 0.001), breathlessness and wheezing in 14% (*n* = 434; min 7.3; max 17; *p* < 0.001), and paroxysmal cough in 15% (*n* = 489; min 4.4; max 19; *p* < 0.001). Diagnosis of bronchial asthma was the highest in Lviv City (4.4% of children), but overall was found in only 2.3% of all participants in the greater Lviv region.

Pollinosis was found in 7% of the children (*n* = 225; min 3.9; max 11; *p* < 0.001), and reddening and itching of the eyes in 3.4% (*n* = 109; min 1.9; max 5.9; *p* < 0.001). It should be noted that the highest percentages of children in a zone with the aforementioned symptoms were found in the Carpathians (11 and 5.9%, respectively).

Signs of urticaria were observed in 18% (*n* = 586; min 17; max 18; *p* = 0.65) of children, with 7 of them (1.2%) experiencing recurrent episodes.

Reactions to the use of cosmetics were recorded in 7% (*n* = 225; min 5.5; max 9.8; *p* = 0.045) of children and manifested with skin symptoms like itching, burning, urticaria, and swelling of lips or eyelids (in 96% of children, *n* = 215). The airways were rarely involved as well as both the skin and airways (2.7% of children, *n* = 6).

Contact dermatitis was reported in 8.8% of children (*n* = 283; min 0.3; max 20; *p* < 0.001). In 97% (*n* = 273) of these cases, the dermatitis was associated with diapers, in 1.1% (*n* = 3) with metal contact (alloys, silver, gold), in 0.4% (*n* = 1) with latex, and in 2.1% (*n* = 6) with other causes (clothes, laundry detergent).

Reactions after using medications were observed in 13% of children (*n* = 418; min 9.1%; max 23%; *p* < 0.001). Allergy to medication was recorded in 10% (*n* = 43), with 40% (*n* = 17) from antibiotics (penicillin or cephalosporin family), 23% (*n* = 10) from non-steroidal anti-inflammatory drugs (paracetamol, ibuprofen), and 37% (*n* = 16) from other medicines (vitamins, anthelmintics, homeopathic and herbal remedies). In 90% (*n* = 375), reactions after medication use were associated with administration of syrup forms and suspensions.

**Figure 4:**
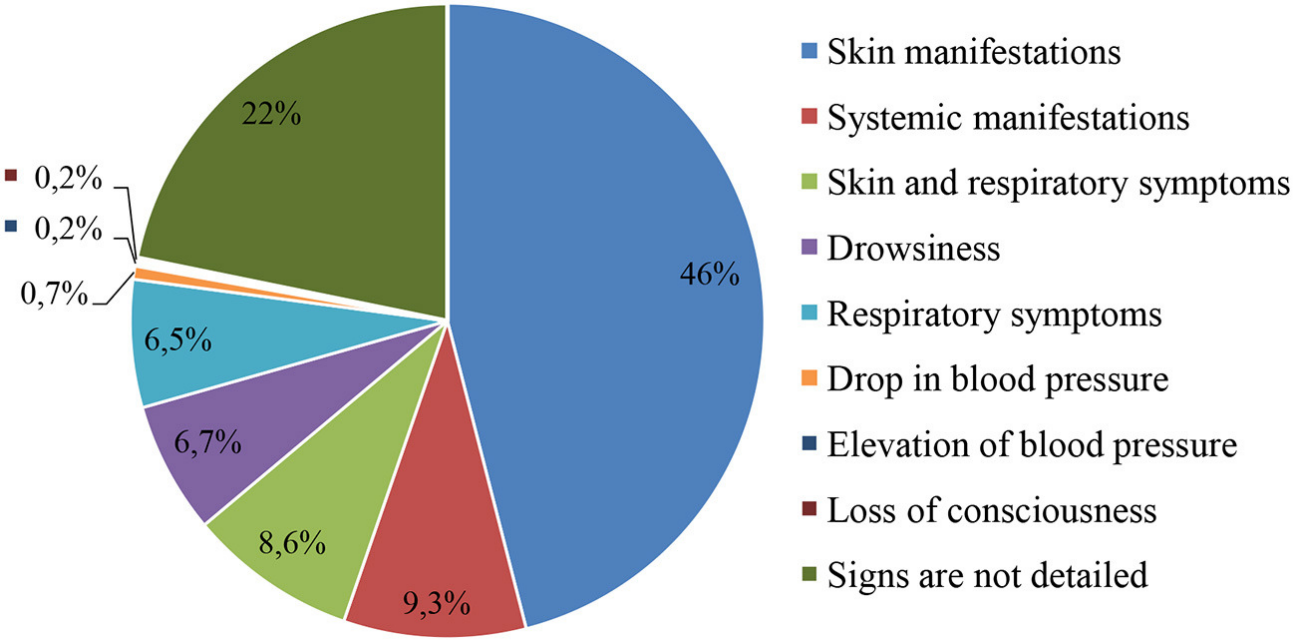
Manifestations of adverse reactions to medications observed in young children.

The different manifestations of adverse reactions after medication use are presented in Figure 4. Development of an anaphylactic reaction due to the use of medications (ceftriaxone, administered intravenously by stream infusion; ibuprofen, oral suspension) was seen in two children.

### Family Medical History and Smoking Status

The responses to part B of the detailed information section of the questionnaire provided information about family medical history and smoking status (Table 6). Gastroduodenal diseases dominated in the family medical history (7.7% of respondents, *n* = 248; min 3.0%; max 14%; *p* < 0.001). Among allergic signs in relatives of the children, pollinosis was the most common (3.8%, *n* = 121; min 0.3%; max 6.8%; *p* = 0.001). The third most common medical condition in family members of children was skin diseases (2.7%, *n* = 87; min 1.5%; max 4.3%; *p* = 0.006). The factor of tobacco smoking by a family member was present in 3.5% of respondents (*n* = 113; min 0.7%; max 7.9%; *p* < 0.001). It should be noted that the lowest percentages of tobacco smoking in the family were recorded in Lowland and Lviv City, while the highest were in the Carpathian foothills and the Carpathians.

**Table 6:** Distribution of children among geographic, climate zones, and influence of family medical history and smoking status^*^.

**Family medical history and smoking status**	**Number (%) of respondents in each zone**					**Total number (%) of respondent**, ***p*** **value**		**Number (%) of respondents with FH**		
	**Forest–grassland**	**Lowland**	**Carpathian foothills**	**Carpathians**	**Lviv City**			**Positive status**	**Negative status**	***p* value**
Bronchial asthma	16 (2.5)	13 (1.9)	25 (3.7)	16 (2.6)	2 (0.3)	72 (2.2)	0.001	22 (31)	787 (25)	0.353
Pollinosis	44 (6.8)	12 (1.8)	30 (4.5)	33 (5.4)	2 (0.3)	121 (3.8)	<0.001	42 (35)	767 (25)	0.018
Urticaria	20 (3.1)	5 (0.7)	19 (2.8)	10 (1.6)	8 (1.3)	62 (1.9)	0.008	19 (31)	790 (25)	0.392
Skin diseases	28 (4.3)	10 (1.5)	21 (3.1)	19 (3.1)	9 (1.5)	87 (2.7)	0.006	26 (30)	783 (25)	0.367
Gastroduodenal diseases	68 (11)	20 (3.0)	59 (8.8)	82 (14)	19 (3.1)	248 (7.7)	0.001	61 (25)	748 (25)	0.888
Tobacco smoking	21 (3.2)	5 (0.7)	30 (4.5)	48 (7.9)	9 (1.5)	113 (3.5)	0.001	32 (28)	777 (25)	0.500

* *Based on analysis of detailed information from the questionnaire (part B)*.

### Environment and Living Conditions

Overall, 58% of respondents were city residents (reflecting 100% in Lviv City, and >50% in two other zones: Forest-Grassland and Lowland). Among all respondents, 64% lived in apartments, 60% in old buildings and 46% in damp buildings (Table 7).

**Table 7:** Comparison of living conditions of children from different geographic and climate zones^*^.

	**Number (%) of respondents in each zone**					**Total number (%) of respondents**, ***P*** **value**		**Number (%) of respondents with FH**		
	**Forest–Grassland**	**Lowland**	**Carpathian foothills**	**Carpathians**	**Lviv City**			**In the indicated geographic and climate zone group**	**In another geographic and climate zone groups**	***P* value**
**Place of residence**										
City	356 (55)	357 (53)	314 (47)	234 (39)	615 (100)	1,876 (58)	<0.001	518 (28)	291 (22)	<0.001
Village	293 (45)	312 (47)	359 (53)	373 (61)	0 (0)	1,337 (42)		291 (22)	518 (28)	<0.001
**Type of dwelling**										
House	192 (30)	254 (38)	308 (46)	349 (58)	58 (9.4)	1,161 (36)	<0.001	256 (22)	553 (27)	0.002
Apartment	457 (70)	416 (62)	365 (54)	258 (43)	557 (91)	2,053 (64)		553 (27)	256 (22)	0.002
**Age of residence**										
New building	272 (42)	287 (43)	197 (29)	310 (51)	231 (38)	1,297 (40)	<0.001	330 (25)	479 (25)	0.802
Old building	377 (58)	383 (57)	476 (71)	297 (49)	384 (62)	1,917 (60)		479 (25)	330 (25)	0.802
**Humidity in residence**										
Dry building	399 (62)	362 (54)	336 (50)	229 (38)	423 (69)	1,749 (54)	<0.001	429 (25)	380 (26)	0.381
Damp building	250 (39)	308 (46)	337 (50)	378 (62)	192 (31)	1,465 (46)		380 (26)	429 (25)	0.381

* *Based on analysis of detailed information from the questionnaire (part C)*.

### Nutritional Habits

Less than one quarter of Lviv City residents consumed homemade food while the percentages of respondents living in the Carpathian foothills and the Carpathians were more than 50% (Table 8). Residents of Lviv City (10%) more often than other respondents visited eateries (cafes and restaurants, including fast food).

**Table 8:** Nutritional habits among children of different geographic and climate zones^*^.

**Nutritional habits**	**Number (%) of respondents in each zone**					**Total number (%) of respondents**, ***P*** **value**		**Number (%) of respondents with FH**		
	**Forest–Grassland**	**Lowland**	**Carpathian foothills**	**Carpathians**	**Lviv City**			**With indicated nutritional habit**	**With another nutritional habits**	***P* value**
Homemade food	258 (40)	235 (35)	401 (60)	361 (60)	142 (23)	1,397 (44)	<0.001	324 (23)	485 (27)	0.026
Purchased food	195 (30)	308 (46)	193 (29)	165 (27)	299 (49)	1,160 (36)	<0.001	274 (24)	535 (26)	0.139
Special baby food^†^	163 (25)	107 (16)	67 (10)	68 (11)	111 (19)	516 (16)	<0.001	170 (33)	639 (24)	<0.001
Dine at eateries ^‡^	33 (5.1)	20 (3.0)	12 (1.8)	13 (2.1)	63 (10)	141 (4.4)	<0.001	41 (29)	768 (25)	0.320

* *Based on analysis of detailed information from the questionnaire (part C)*.

† *Semi-processed food; bottled products for children*.

‡ *Cafes and restaurants, including fast food*.

## Discussion

In this cross-sectional study the prevalence of food hypersensivity in 0–3-year-old children living in the Lviv region in Ukraine reported by their parents was 25%.

Sensitization to cow's milk was the most common in young children with food hypersensitivity and the peak incidence of milk hypersensitivity was observed at the age of 1–2 years. Other top causative foods for food hypersensitivity were hen's egg and wheat.

The study of food hypersensitivity in Ukraine is a rather urgent issue, since there are only a few published reports to date. Data about prevalence of food hypersensitivity in 0–3-year-old children is absent in our country and limited in the world. Furthermore, it is quite problematic to compare our data with the data of other researchers, first of all due to the fact that a uniform standardized questionnaire was not used, but also because there were different approaches to sampling and different age groups were studied. In south-eastern Finland the rate of food hypersensitivity reported by parents for their children aged 1–4 years was slightly lower (21%) (13). However, in the United Kingdom according to the data obtained from a cohort born on the Isle of Wight, food-related adverse reactions in children before the age of 3 years were reported by 33.7% of parents (14). The results of a cross-sectional study in which all children from 1 month to 5 years in Uberlândia (Brazil) were enrolled showed that according to the parents' report, food hypersensitivity was present in 23.5% of infants and 17.6% of preschoolers. However, it should be noted that in this study the response rate was <50% (15). To determine the prevalence and regional differences of food hypersensitivity, nine European countries were involved in the EuroPrevall birth cohort study which lasted for 5 years. Standardized questionnaires were used. It has been found that the rate of food hypersensitivity reported by parents fluctuated widely from 5 to 30% (16). Each nation is unique, differing in genetic peculiarities, epigenetic features and lifestyles. Statistics from one country cannot be the same as in another, since there are different cultures, eating habits, nutritional choices, risk factors, habits, living conditions, ecology, and climate.

Milk and egg are the most common foods linked with hypersensitivity in children up to 3 years (14, 16).

According to a survey of parents of infants and toddlers with skin symptoms living in the Zaporizhzhia region of Ukraine, sensitivity to milk and eggs were mentioned by 31 and 12%, respectively.

It is important to note that the rate of food hypersensitivity to an unspecified causal product was 39.8% in a study by Pakholchuk and Nedelska (17), similar to the rate of 34% that we found in the Carpathians. In Brazil, parents reported the rate of food hypersensitivity to cow's milk was higher in infants (52.8%) than in preschoolers (42.7%) (15). Milk hypersensitivity was higher among other products in Finland, but its rate was only 13% (13).

The high rate of hypersensitivity to egg in the first 2 years of life was also reported in several studies (17, 18). Moreover, Salehi et al. stated that hypersensitivity to chicken eggs was dominant in children under 2 years of age, whereas in the age group of 2–5 years, sensitivity to milk prevailed (19). It was also acknowledged that egg was the most important food allergen in children with atopic dermatitis under 2 years old (20). The clinical study results of the prevalence of food allergy among kindergarten children in Samsun (Turkey) show that the most common allergenic foods reported by parents of 3–5-year-old children were hen's egg (25.3%) followed by chocolate, strawberry and milk (18).

Undoubtedly, the structure of sensitization to different groups of allergens changes with age (21).

Currently, researchers in many countries are studying the incidence of food hypersensitivity depending on individual possibilities and influences of different factors (22, 23). The number of children suffering from a certain type of food hypersensitivity is constantly increasing, which can be explained by ecology, living conditions, lifestyle, nutritional habits, and new technologies for food processing, as well as the common use of food additives, dyes, preservatives, and flavoring agents which can be the cause of the development of allergic conditions (24).

The sensitization to certain food allergens depends on many factors. Not surprisingly, residents living in different geographic and climate zones have their own nutrition peculiarities which result in a higher frequency of adverse reactions to diverse products (25). Particularly in the Carpathians, there is higher hypersensitivity to fish and honey than in other regions, which is likely associated with frequent presence of these products in a child's diet. Hypersensitivity to soy was detected only in Lviv City residents; however, it is a hidden ingredient in many other products (confectionery, meat products, sauces, and semi-processed food). This situation indicates that patients' case histories should be recorded thoroughly with onset of symptoms and that additional examinations and laboratory tests should be performed in medical establishments.

Geographic remoteness from specialized medical institutions and lack of diagnostic access in rural areas (the Carpathians) compared to urban regions (Lviv) make distinguishable differences in the diagnosis and treatment of food hypersensitivities.

The data based on parentally reported past or present severe diseases show that the most common were pulmonological and otolaryngological illnesses like pneumonia, laryngitis, obstructive bronchitis, otitis, and tonsillitis. These diseases were usually due to the influence of a virus or to the combined influence of a bacterial agent following a past viral infection. These situations are caused by a number of factors, such as the immaturity of the immune system as well as anatomical and physiological peculiarities of different organs and systems in children.

The explanation for the highest percentages of respondents with symptoms of pollinosis that were observed in the Carpathians must consider that children in this zone are surrounded by lots of different plants in nature, and thus signs of seasonal rhinitis and conjunctivitis may occur.

The allergic reactions after administration of syrup forms and suspensions in some cases are not associated with the medicine itself, but rather with its diluents. Food additives can give taste, aroma, density, homogeneity of structure and long shelf-life to medicines. They are marked as “E” index and indicated as additional substances of the medicine composition.

This investigation is appropriate for understanding the problem of food hypersensitivity in the Lviv region. It is a novel study in Ukraine and Eastern Europe and offers significant practical sense for the formation of recommendations for children with food hypersensitivity and food allergy.

There are several limitations of our study. First, the study did not cover all children of this age group born and living in a certain geographic and climatic zone of the Lviv region because some children do not attend pre-schools as well as some parents ignore routine health visits. Second, the data provided by the parents of young children is based on their perception of food and sensitization to main food allergens. Therefore, the true rate of food hypersensitivity may be lower than reported by parents.

Finally, our data represent only the Lviv region of Ukraine, however the rate of food hypersensitivity in other regions may differ, which in turn will determine the frequency of food hypersensitivity in Ukraine as a whole.

Our food hypersensitivity investigation in the Lviv region showed that this theme needs elaboration. The questionnaires should be administered uniformly in other regions of Ukraine. Such administration will result in the generation of a national database which can be compared to the data from other countries.

## Conclusion

This study provides the first population-based epidemiological information related to food hypersensitivity in young children (age: 0–3 years) living in the Lviv region of Ukraine. It can be assumed that the acquired data are consistent with the data of other researchers. Some peculiarities found in four geographic and climate zones as well as in Lviv City have considerable practical sense for formulation of recommendations for children with food hypersensitivity. Further clinical studies are required to investigate the structure of food allergens in children with reported food hypersensitivity.

## Data Availability Statement

The original contributions presented in the study are included in the article/supplementary material, further inquiries can be directed to the corresponding author.

## Ethics Statement

The studies involving human participants were reviewed and approved by Communal Nonprofit Enterprise City Children's Clinical Hospital of Lviv, Allergy Department, Ukraine. Written informed consent to participate in this study was provided by the participants' legal guardian/next of kin.

## Author Contributions

OM: investigation, writing, validation, data curation, and original draft. LB: conceptualization, methodology, and supervision. OBo: investigation and data interpretation. TG and OK: software management and data analysis. AM, OBe, and OS: investigation. SV: concept revisions and editing. All authors contributed equally to the manuscript, read, and approved the final version of the manuscript.

## Funding

This research was supported by the Association for Regional Cooperation in the Fields of Health, Science and Technology (RECOOP HST Association) RECOOP Grant #009, 2019–2020.

## Conflict of Interest

The authors declare that the research was conducted in the absence of any commercial or financial relationships that could be construed as a potential conflict of interest.

## Publisher's Note

All claims expressed in this article are solely those of the authors and do not necessarily represent those of their affiliated organizations, or those of the publisher, the editors and the reviewers. Any product that may be evaluated in this article, or claim that may be made by its manufacturer, is not guaranteed or endorsed by the publisher.
